# SICQ Coping and the Health-Related Quality of Life and Recovery of Critically Ill ICU Patients: A Prospective Cohort Study

**DOI:** 10.1016/j.chest.2021.06.033

**Published:** 2021-06-26

**Authors:** Edwin J. Boezeman, José G.M. Hofhuis, Christopher E. Cox, Reinout E. de Vries, Peter E. Spronk

**Affiliations:** a Section Social, Economic and Organizational Psychology, Faculty of Social and Behavioral Sciences, Leiden University, Leiden, The Netherlands; b Department of Intensive Care Medicine, Gelre Hospital Apeldoorn, Apeldoorn, The Netherlands; c Department of Medicine, Duke University, Durham NC; d Department of Experimental and Applied Psychology, Vrije Universiteit Amsterdam, Amsterdam, The Netherlands; e Department of Intensive Care Medicine, Academic Medical Center Amsterdam, Amsterdam, The Netherlands; f Expertise center for Intensive care Rehabilitation Apeldoorn (ExpIRA), The Netherlands

**Keywords:** coping skills, critical care, ICU, quality of life, APACHE, Acute Physiology and Chronic Health Evaluation III, HR, hazards ratio, HRQoL, health-related quality of life, SF-12, Short Form 12-item, SICQ, Sickness Insight in Coping Questionnaire

## Abstract

**Background:**

The coping styles of the Sickness Insight in Coping Questionnaire (SICQ; positivism, redefinition, toughness, fighting spirit, nonacceptance) may affect the health and recovery of hospitalized critically ill patients.

**Research Question:**

Do the SICQ coping styles of hospitalized critically ill patients relate to the patients health-related quality of life (HRQoL) and recovery?

**Study Design and Methods:**

A prospective cohort study was conducted in a single university-affiliated Dutch hospital. Participants were critically ill adult patients admitted to a mixed medical-surgical ICU (start: n = 417; pre-ICU: n = 391; hospital discharge: n = 350; 3-month follow-up: n = 318; 6-month follow-up: n = 308; 12-month follow-up: n = 285). Coping was recorded with the SICQ pre-ICU and at discharge. HRQoL was measured with the SF-12 pre-ICU, at discharge, and 3, 6, and 12 months after discharge. Indicators of recovery were ICU and hospital length of stay, discharge disposition, and mortality. Correlation and regression analyses were used for data analysis.

**Results:**

Positivism (*r* = 0.28-0.51), fighting spirit (*r* = 0.14-0.35), and redefinition (*r* = 0.12-0.23) associated significantly (*P* < .05) with mental HRQoL after discharge. Furthermore, positivism associated positively (*P* < .01) with physical HRQoL (*r* = 0.17-0.26) after discharge. Increase in positivism (*r* = 0.13), redefinition (*r* = 0.13), and toughness (*r* = 0.13) across the period of hospitalization associated positively (*P* ≤ .05) with mental HRQoL at discharge. Pre-ICU positivism associated with hospital length of stay (*ρ* = −.21, *P* ≤ .05) and hazard for death (HR = 0.57, *P* < .01) and had a unidirectional effect on mental HRQoL (β = .30, *P* < .001).

**Interpretation:**

SICQ coping is associated with long-term mental HRQoL, hospital length of stay, and hazard for death among hospitalized critically ill patients.

## Graphical Abstract

**Figure:**
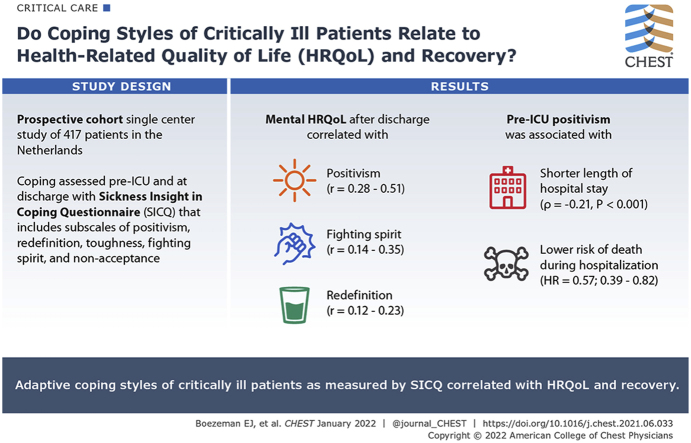

**Take-home Points**

**Study Question:** Do the SICQ coping styles of hospitalized critically ill patients relate to the patients health-related quality of life (HRQoL) and recovery?

**Results:** The SICQ coping styles of the hospitalized critically ill patients correlated with the patients HRQoL and recovery. The SICQ positivism of the hospitalized critically ill patients most consistently associated with the patients’ HRQoL and recovery.

**Interpretation:** In the caretaking of hospitalized critically ill patients, assessing and addressing the adaptive coping of these patients is relevant.

Patients treated in an ICU commonly experience psychological distress for a number of reasons, including discomfort due to invasive medical treatments, the unfamiliar environment, and intrusive thoughts about mortality.^1^, ^2^, ^3^, ^4^, ^5^, ^6^ As a result of this distress, patients may report decreased health-related quality of life (HRQoL) and impaired recovery during the ICU stay and after discharge.^7^, ^8^, ^9^ Adaptive coping helps in decreasing psychological distress, and in general and patient populations adaptive coping positively affects mental health and HRQoL.^10^, ^11^, ^12^, ^13^ Accordingly, researchers have begun to address the coping styles of ICU survivors,^14^, ^15^, ^16^ family members of ICU patients,^17^ ICU staff members,^18^ and hospitalized ICU patients.^19^^,^^20^ However, research in this area is still underdeveloped. The Sickness Insight in Coping Questionnaire (SICQ) is an instrument that measures coping styles of particular relevance to the experience of serious illness during and after hospitalization, including positivism (ie, having a positive attitude), redefinition (ie, seeing advantages of the medical situation such as personal growth), toughness, fighting spirit, and nonacceptance (of the current medical condition and its possible outcome).^20^ A previous SICQ study has focused on its measurement properties and found good structural validity, good patient-proxy agreement, and high reliability of its subscales.^20^ However, whether SICQ-measured coping styles are associated with ICU patients’ HRQoL and recovery remains unclear. Therefore, the aim of the current research was to examine whether the patient-reported SICQ-assessed (ie, assessment pre-ICU and after discharge) coping scores of severely ill patients relate to HRQoL and recovery As a supplementary analysis, whether the pre-ICU proxy-reported SICQ-assessed coping of critically ill patients had a role in the patient-reported HRQoL and recovery was examined.

## Methods

### Ethical Consent

After informing the Gelre Hospital Medical Ethics Review Board about the research and obtaining oral consent for the research participants, a waiver for this study was obtained from the Gelre Hospital Medical Ethics Review Board (reference number TCO-12.17) that judged that this study was not subject to the Medical Research Involving Human Subjects Act.

### Study Design and Setting

A prospective observational cohort study with 1-year follow-up was conducted. The data of the current research were collected in the context of a cohort study on the HRQoL of ICU patients.^21^ The study was conducted in the Gelre Hospitals, a Dutch 650-bed university-affiliated teaching hospital in Apeldoorn with a 14-bed medical-surgical ICU. The research consisted of five measurement occasions; patient data were collected pre-ICU, at the moment of hospital discharge, and 3, 6, and 12 months after hospital discharge.

### Participants and Procedures

The participants were adult patients admitted to the ICU. Patients with language barriers or cognitive disorders were excluded from participation.^22^ After oral consent (the consent was later recorded in writing in the patient’s file), the patients able to complete a questionnaire (eg, those not on mechanical ventilation) and close family members (proxies) of the patients were asked to complete the first questionnaire recording information about the patients’ demographic characteristics, coping, and HRQoL. Close family members of patients were included in the research as proxies, because some ICU patients were not able to complete a questionnaire at the time of admission. Proxies had to be in close contact with the patient on a regular basis, and they were asked to answer on behalf of the patient and mark the statement that best described the patient’s state of health and perceived coping style in the last 4 weeks before the admission. This method proved to be a reliable way for assessment as shown in previous studies.^20^, ^21^, ^22^ At the moment of hospital discharge, the included patients (ICU survivors) completed the second questionnaire of the research that recorded patient coping and HRQoL at the moment of hospital discharge. Three (ie, third questionnaire), 6 (ie, fourth questionnaire), and 12 (ie, fifth questionnaire) months after hospital discharge, the patients completed the questionnaire that recorded their HRQoL at the time of the measurement. The follow-up questionnaires were completed by the participants via telephone. The telephone follow-up data collection was organized and done by study team member J. H.

### Measurements

The pre-ICU proxy questionnaire recorded the demographic characteristics of the study participants. Study staff used the hospital patient information monitoring system to abstract severity of illness (ie, APACHE—Acute Physiology and Chronic Health Evaluation III score) at the time of ICU admission, hospital and ICU length of stay, patient location after resignation from the hospital (ie, nursing home or rehabilitation center vs home), and 12-month mortality.

The SICQ^20^ was used to assess the patients’ coping styles pre-ICU and at the moment of hospital discharge. The SICQ has five subscales, each corresponding to a specific coping style, and records positivism, redefinition, toughness, fighting spirit, and nonacceptance. Each SICQ subscale has three items, and responses to the items are recorded on a 5-point Likert scale. A higher SICQ score suggests more adaptive coping. The Short Form 12-item (SF-12) instrument,^23^ a shortened version of the SF-36,^24^ was used to record the mental and physical HRQoL of the patients. The SF-12 was administered to the patients pre-ICU, at the moment of hospital discharge, and 3, 6, and 12 months after hospital discharge. The SF-12 yields a mental HRQoL score (six items) and a physical HRQoL score (six items), with higher scores indicating a better quality of life.^25^

## Analysis

The correlation analyses, Cox regression survival analyses, and logistic regression analyses were conducted with the IBM SPSS statistical software.^26^ The EQS statistical software was used for conducting cross-lagged path analysis.^27^

Multicollinearity between domains of the SICQ was absent (*r* < 0.75) based on Pearson correlation tests. We used Pearson correlations to assess the associations between patients’ SICQ coping scores (recorded before admission to the ICU as well as directly after hospital discharge) and HRQoL (at hospital discharge and 3, 6, and 12 months after hospital discharge). Furthermore, Spearman correlation analysis was used to examine the relations between the patients’ pre-ICU SICQ scores and skewed length of stay. In addition, partial correlation analyses were conducted to obtain further insight in these relations when controlling for the severity of illness—score (APACHE III), age, and sex (0 = male, 1 = female). Pearson correlation analysis was used to examine whether an increase in patients’ SICQ coping scores from the pre-ICU to hospital discharge period was associated with the HRQoL at the moment of hospital discharge and in the months after discharge from the hospital. According to Funder and Ozer,^28^ correlation coefficients are of small size in case of a value below .20, of medium size in case of a value between .20 and .30, and of large size in case of a value of .30 or higher.^28^ Cox regression survival analysis was used to examine whether the pre-ICU coping predicted hazard for death during study follow-up. Logistic regression analyses were conducted to examine whether the patients’ pre-ICU SICQ scores were associated with patient discharge disposition (ie, nursing home or rehabilitation center vs home). Furthermore, after the repeated measurement of variables, cross-lagged path analysis was used to examine whether the presumed predictors more strongly affected outcomes than vice versa. Specifically, cross-lagged path analysis^29^^,^^30^ using summary scores was used to examine whether the SICQ scores affected the patients’ HRQoL in a unidirectional way (ie, the SICQ-coping score more strongly predicted HRQoL than HRQoL predicted SICQ-coping). Per recommendations for conducting cross-lagged path analysis in EQS, the variables recorded at discharge were all correlated to each other by allowing their error terms to correlate.^30^ Omnibus cutoff criteria were used to evaluate the overall model fit of the crosslagged path model (ie, nonsignificant χ^2^ test, the NNFI and CFI values ≥ 0.95, RMSEA < 0.05).^31^ The Supplementary Materials include the results on the associations between pre-ICU proxy rated SICQ coping and outcomes, including patients’ HRQoL, length of stay, location of stay after discharge, and mortality.

**Figure 1:**
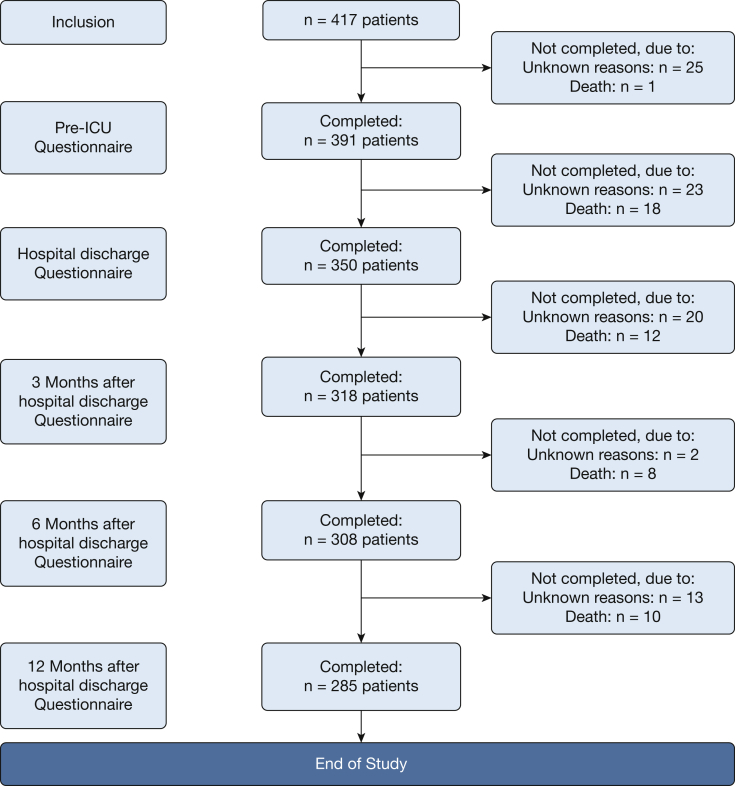
Flow diagram of the study and its participants.

## Results

### Sample Overview

Of 712 ICU admissions, a total of 295 patients were excluded, and 417 patients (184 women and 233 men) provided consent and were included. The patients’ average age was 65.7 years, and 55.9% were male (Table 1). Figure 1 shows the number of patients at each study time point. In addition, 133 family members (patient proxies) provided information about the coping of the patients.

**Table 1:** Demographic Characteristics of Patients

Category	Scores
Age, mean (SD, range)	65.7 (13.7; 23-92)
Men, No. (%)	233 (55.9%)
Women, No. (%)	184 (44.1%)
ICU length of stay in days, mean (SD; range)	3.8 (6.3; 0-67)
Hospital length of stay in days, mean (SD; range)	16 (17.8; 2-187)
Acute Physiology and Chronic Health	
Evaluation score (APACHE), mean (SD; range)	16.5 (6.3; 2-43)
Simplified Acute Physiology Score	
(SAPS), mean (SD; range)	34.7 (13.9; 0-85)
Type of admission	
Elective surgical, No. (%)	225 (54%)
Acute surgical, No. (%)	80 (19.2%)
Medical, No. (%)	107 (25.7%)
Unknown (missing values), No. (%)	5 (1.2%)
Infection when admitted to the ICU, yes; No. (%)	107 (25.7%)
Mechanical ventilation when admitted to the ICU, yes; No. (%)	166 (39.8%)
Mechanical ventilation in first 24 hours after admission to the ICU*,* yes; No. (%)	217 (52%)
After hospital discharge, patient was discharged to:	
Home, No. (%)	295 (70.7%)
Rehabilitation center, No. (%)	13 (3.1%)
Nursing home, No. (%)	51 (12.2%)
Other location, No. (%)	23 (5.5%)
Unknown location (missing value), No. (%)	13 (3.1%)
Patient deceased during hospital stay, No. (%)	22 (5.3%)

### Coping Scores and HRQoL Correlations

The patients’ positivism (*r* = .28 to .51) and fighting spirit (*r* = .14 to .35), recorded pre-ICU and at discharge from the hospital, associated significantly (*P* < .05) with the patients’ mental SF-12 scores in the months after discharge (Table 2). Furthermore, the pre-ICU redefinition associated significantly (*r* = 0.12-0.13) with the SF12 mental component scores in the months after discharge (Table 2). Furthermore, positivism correlated positively and significantly (*r* = 0.17-0.26, *P* < .05) with the SF12 physical component scores throughout follow-up (Table 3). Although significant (*P* < .05) positive correlations were observed between toughness and SF12 scores, nonacceptance was not found to be clearly associated with SF-12 mental or physical component scores (Tables 2 and 3). Increases in patients’ positivism, redefinition, and toughness from the pre-ICU to postdischarge period correlated significantly (*P* < .05) with the patients’ SF-12 mental component scores at hospital discharge (Table 4). Correlations between proxy-recorded SICQcoping and patient HRQoL are shown in e-Table 1, which demonstrates that the pre-ICU proxy-reported positivism was associated with patient-reported mental HRQoL, whereas the proxy-reported pre-ICU redefinition was significantly associated with the patient-reported physical HRQoL.

**Table 2:** Correlations Between SICQ-Coping and ICU Patients’ Mental HRQoL

SICQ Coping Styles	Mental Quality of Life, Recorded					
	SICQ SCORE		At Hospital Discharge (n = 350)	3 mos after discharge (n = 318)	6 mos after discharge (n = 308)	12 mos after discharge (n = 285)
	M (SD)	Range				
SICQ-coping recorded pre-ICU						
Positivism	3.90 (.87)	1.33-5.00	.38^a^ (.37^b^;.35^b^;. 37^b^)	.38^a^ (.37^b^;.37^b^; 35^b^)	.35^a^ (.33^b^; .33^b^;33^b^)	.30^a^ (.27^b^;.27^b^;.27^b^)
Redefinition	2.66 (.88)	1.00-4.67	.12^b^ (.12^b^;.10;.12^b^)	.12^b^ (.12^b^;.11;.12^b^)	.12^b^ (.12^b^;.11;.12^b^)	.13^b^ (.14^b^;.12^b^;.14^b^)
Toughness	3.75 (.81)	1.00-5.00	.22^a^ (.24^b^;.20^b^;.22^b^)	.11 (.09;.10;.11)	.08 (.06;.06;.08)	.04 (.03;.02;.04)
Fighting spirit	4.39 (.71)	1.67-5.00	.19^a^ (.20^b^;.17^b^;.19^b^)	.14^b^ (.15^b^;.13^b^;.13^b^)	.15^b^ (.15^b^;.14^b^;.15^b^)	.16^b^ (.16^b^;.15^b^;.16^b^)
Nonacceptance	2.38 (.84)	1.00-5.00	−.04 (−.05; −.07; −.04)	.04 (.03; .01; .04)	−.01 (−.02; −.03; −.01)	−.04 (−.04; −.06; −.05)
SICQ-coping recorded at discharge						
Positivism	4.08 (.83)	1.00-5.00	.51^a^ (.49^b^; .49^b^; .50^b^)	.31^a^ (.30^b^; .29^b^;.30^b^)	.31^a^ (.30^b^; .29^b^; .29^b^)	.28^a^ (.28^b^; .26^b^; .27^b^)
Redefinition	2.97 (.87)	1.00-5.00	.23^a^ (.23^b^; .21^b^; .22^b^)	.11 (.11; .09; .11)	.11 (.11; .09; .11)	.11 (.13^b^; .10^b^;.11)
Toughness	3.80 (.78)	1.00-5.00	.29^a^ (.31^b^; .27^b^;. 29^b^)	.17^c^ (.16^b^; .16^b^; .17^b^)	.11 (.11; .09; .11)	.08 (.08; .06^b^; .08)
Fighting spirit	4.37 (.78)	1.00-5.00	.35^a^ (.36^b^; .34^b^; .36^b^)	.25^a^ (.26^b^; .24^b^; .27^b^)	.23^a^ (.23^b^;.21^b^;.24^b^)	.26^a^ (.26^b^;.25^b^;.28^b^)
Nonacceptance	2.46 (.83)	1.00-5.00	−.08 (−.08; −.10; −.08)	−.03 (−.03; −.04; −.03)	−.10 (−.11; −.11; −.10)	−.12^b^ (−.12^b^; −.13^b^; −.12^b^)

Coefficients between parentheses are partial correlation coefficients controlling for the severity of illness—score (APACHE), age, and sex (0 = male, 1 = female).

a *P* < .001.

b *P* < .01.

c *P* < .05.

**Table 3:** Correlations Between SICQ-Coping and ICU-Patients’ Physical HRQoL

SICQ Coping Styles	Physical Quality of Life, Recorded:			
	At Hospital Discharge (n = 350)	3 Mos After Discharge (n = 318)	6 Mos After Discharge (n = 308)	12 Mos After Discharge (n = 285)
SICQ-coping recorded pre-ICU				
Positivism	.17^a^ (.14^b^; .11^b^; .15^b^)	.25^a^ (.22^b^; .20^b^; .25^b^)	.25^a^ (.21^b^;.20^b^;.24^b^)	.17^a^ (.13^b^; .10; .13^b^)
Redefinition	.04 (.04; .00; .03)	.10 (.10; .07; .10)	.17^a^ (.18^b^; .15^b^; .17^b^)	.11 (.11; .10; .12)
Toughness	.16^a^ (.16^b^; .14^b^; .16)	.04 (.03; .02; .04)	.02 (.01; −.01; .02)	.02 (.02; −.02; .02)
Fighting spirit	.06 (.06; .05; .06)	.11 (.10; .10; .11)	.14^b^ (.13^b^; .13^b^; .14^b^)	.06 (.06; .04; .06)
Nonacceptance	.09 (.08; .06; .09)	.02 (.00; −.01; .02)	−.01 (−.02; −.05; −.02)	−.03 (−.03; −.06; −.04)
SICQ-coping recorded at discharge				
Positivism	.26^c^ (.23^b^; .22^b^; .24^b^)	.20^c^ (.18^b^; .17^b^; .20^b^)	.21^c^ (.18^b^; .18^b^; .20^b^)	.20^c^ (.18^b^; .16^b^; .18^b^)
Redefinition	.16^a^ (.17^b^; .13^b^; .15^b^)	.05 (.06; .02; .05)	.14^b^ (.15^b^; .12^b^; .14^b^)	.17^a^ (.18^b^; .14^b^; .17^b^)
Toughness	.13^b^ (.12^b^; .09; .12^b^)	.04 (.03; .00; .04)	.11 (.11; .07; .10)	.11 (.11; .06; .11)
Fighting spirit	.02 (.00; −.01; .02)	.11 (.10; .09; .12^b^)	.18^a^ (.16^b^; .16^b^; .18^b^)	.14^b^ (.14^b^; .11; .16^b^)
Nonacceptance	.00 (.00; −.02; .00)	−.06 (−.07; −.08; −.06)	−.08 (−.08; −.10; −.07)	−.14^b^ (−.13^b^; −.16^b^; −.14^b^)

Coefficients between parentheses are partial correlation coefficients controlling for the severity of illness—score (APACHE), age, and sex (0 = male, 1 = female).

a *P* < .01.

b *P* < .05.

c *P* < .001.

**Table 4:** Correlations Between Change in SICQ-Coping Over Time (Pre-ICU to Hospital Discharge Period) and Quality of Life

Quality of Life	Change in SICQ-Coping in ‘Pre-ICU to Hospital-Discharge’—Period				
	*Δ*Positivism	*Δ*Redefinition	*Δ*Toughness	*Δ*Fighting Spirit	*Δ*Nonacceptance
Mental quality of life					
At hospital discharge	.13^a^	.12^a^	.12^a^	.11	−.06
3 mos after discharge	−.06	.01	.08	.11	−.10
6 mos after discharge	−.04	.01	.03	.11	−.12^a^
12 mos after discharge	−.01	-.00	.06	.19^b^	−.12^a^
Physical quality of life					
At hospital discharge	.08	.12^a^	−.03	−.13^a^	−.12^a^
3 mos after discharge	−.07	−.06	−.02	.04	−.11
6 mos after discharge	−.03	−.02	.11	.12^a^	.09
12 mos after discharge	.02	.05	.08	.07	−.16^b^

A positive significant correlation suggests that an increase in the domain of SICQ-coping in the ‘pre-ICU to hospital discharge period’ (ie, over time) corresponds with an increase in HRQoL, whereas a negative significant correlation suggests that a decrease in the domain of SICQ-coping in the time period corresponds with a decrease in HRQoL.

a *P* < .05.

b *P* < .01.

### Trajectory to Recovery

The patients’ positivism recorded pre-ICU correlated significantly and negatively with the hospital (ρ = −.21, *P* < .001) and ICU (ρ = −.16, *P* < .001) length of stay (e-Table 2). e-Table 2 shows that the patients’ pre-ICU positivism (ρ = −.29, *P* < .001) and redefinition (ρ = −.25, *P* < .001) as rated by proxy associated negatively with the patients’ hospital length of stay. The Cox regression survival analysis showed that only pre-ICU positivism (B = −.57, *P* < .01, hazards ratio [HR] = .57, HR CI 95% = .39-.82) decreased the hazard for death in the 12 months after admission to the hospital (e-Table 3). Furthermore, logistic regression analysis initially demonstrated that only the pre-ICU positivism determined the patients’ location after hospital discharge (e-Table 4), but this effect of positivism became nonsignificant when control variables (APACHE-3, infection when admitted to the ICU [no/yes], mechanical ventilation when admitted to the ICU [no/yes], and type of admission the ICU [unplanned/planned]) were added as predictors in the logistic regression analysis. e-Table 4 also shows the associations of proxy-completed SICQ scores with discharge disposition. Furthermore, pre-ICU patient-reported posivism and the pre-ICU proxy-reported positivism each have a significant role in hazard for death for critically ill patients (e-Table 3).

### Directionality of Effects

**Figure 2:**
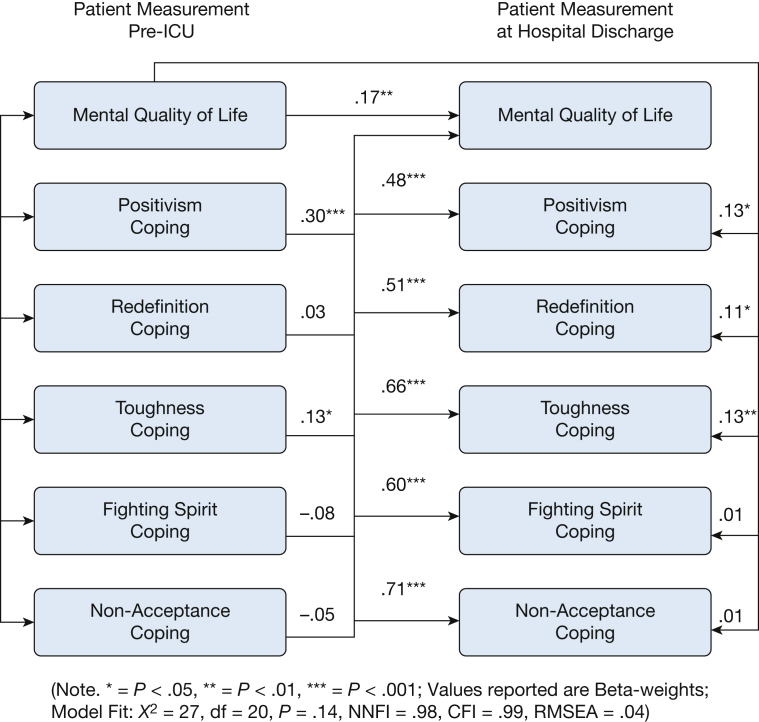
Crosslagged path analysis results (^∗^*P* < .05, ^∗∗^*P* < .01, ^∗∗∗^*P* < .001); Model fit and standardized beta-weights relevant for interpretation of crosslagged effects are shown. Not depicted is that the variables recorded at discharge were all correlated to each other via error term correlations.

The cross-lagged path analysis demonstrated a good model fit (χ^*2*^ = 27, *df* = 20, *P* = .14, Non-Normed Fit Index (NNFI) = .98, Comparative Fit Index (CFI) = .99, Root-Mean-Square-of-Approximation (RMSEA) = .04). This analysis showed that patients’ positivism coping style was the only factor directionally associated with the SF12 mental HRQoL (Fig 2). Pre-ICU recorded positivism strongly predicted the positivism (β = .48, *P* < .001) and SF12 mental HRQoL (β = .30, *P* < .001) recorded at the moment of hospital discharge. Additionally, pre-ICU recorded SF12 mental HRQoL was associated less strongly (β = .13, *P* < .05) with the positivism recorded at hospital discharge. These results suggest that positivism is an unidirectional predictor of mental HRQoL.

## Discussion

This large prospective cohort study found the SICQ adaptive coping styles to be relevant to the patients’ health-related quality of life HRQoL and long-term outcomes. Specifically, positivism was associated with a shorter length of hospital stay and lower risk of death during hospitalization. We also found that pre-ICU proxy ratings of patients’ adaptive coping were associated with patient HRQoL, therefore supporting the validity of proxy completion of the SICQ.

Previously, researchers applied the Coping Status A instrument to neurological ICU patients, finding that coping strategies such as relaxation, assertiveness, and disputing maladaptive thoughts were associated with fewer symptoms of anxiety, depression, and posttraumatic stress disorder.^19^ Other work showed that a psychological intervention that helped critically ill patients cope with the severe medical condition and medical procedures contributes to the patients’ quality of life.^7^ Furthermore, a coping skills training intervention was found to reduce symptoms of depression among ICU survivors with elevated levels of baseline distress.^15^ However, few studies have rigorously examined critically ill patients’ coping styles or how these may impact the health, HRQoL, and recovery of such patients.

The current study represents a contribution to the literature because it addressed the epidemiology of adaptive coping among hospitalized critically ill patients *and* it demonstrated associations of adaptive coping styles with HRQoL and recovery. Previous research showed that negative emotion-focused coping decreases the mental HRQoL of ICU survivors,^14^ whereas mastery coping contributes to the HRQoL of ICU survivors.^16^ Our finding that distinct SICQ coping styles affect the patients’ post-discharge HRQoL may help in improving recovery from critical illness. Accordingly, this research contributes the insight that the way critically ill patients cope with their severe medical condition during their stay at the hospital and ICU has a role in HRQoL and recovery after discharge, and this insight may help researchers and ICU staff members in understanding the post-discharge HRQoL and recovery of ICU survivors.

Our findings also highlight novel approaches for intervention timing and targeting. Screening for low pre-ICU proxy-reported adaptive coping of patients may allow ICU staff members to anticipate that the health condition of certain patients may suffer without intervention and record this in bedside diaries.^32^ Previous work showed that bedside education about coping, plus counseling and assistance to prevent relapse in coping, may prove effective in instilling coping behaviors in ICU patients who are awake and able to communicate.^7^ In line with previous work,^13^ a patient-tailored coping skills training program about SICQ coping may be delivered to ICU survivors in the first week after hospital discharge via mobile apps, by phone, or via more traditional means (eg, a self-help course book), to instill SICQ coping. At the same time, there are limitations worth considering. This was a single-center study conducted among a sample of predominantly western Dutch patients, and thus the findings may not apply to patients in other—nonwestern—settings. Furthermore, the current study, for instance, was not a randomized controlled trial, and thus it only represents a first step in understanding the role of the SICQ coping styles in the health, HRQoL, and recovery of critically ill patients. In addition, the current study did not examine whether adaptive coping as recorded with the SICQ, for instance, decreases the distress and anxiety of hospitalized critically ill patients. Accordingly, new research is needed to examine whether SICQ coping styles affect other types of outcomes among patients, to test the effectiveness of interventions that may instill SICQ coping in patients and to address potential individual and cultural differences in SICQ coping of patients.

Finally, the SICQ is a survey instrument for assessing, characterizing, and likely examining the effectiveness of interventions that aim to strengthen the psychological coping of critically ill patients during hospitalization. In the current research, associations were observed between SICQ coping styles recorded during hospitalization and HRQoL patient scores as recorded during and after hospitalization. Future research is needed to investigate how the SICQ can be applied clinically to understand and enhance patients’ adaptive coping. For instance, by applying the SICQ at different moments in the patient journey of critically ill patients, new insights about psychological coping and its effects may be obtained. This may reveal that the intensity of the different SICQ coping styles may vary temporally during recovery. For instance, critically ill patients may be more inclined to use fighting spirit and toughness while undergoing intensive treatment in the ICU and more inclined to use redefinition during recuperation after intensive treatment at the ICU. Furthermore, examining whether discharged patients continue to use, and value, specific SICQ coping styles over time, and how these behaviors may impact long-term health and recovery, is likely important. The SICQ includes coping concepts that previously have been found helpful to discharged critically ill patients (eg, positivism, redefinition), but also coping concepts (eg, fighting spirit, toughness, nonacceptance) that may be more helpful to discharged patients and their health and recovery than may at first be expected. Indeed, after discharge and later in the process of recovery, patients may struggle to overcome barriers (eg, decreased ability to participate in social activities such as work) that may only be removed by adaptive coping in terms of fighting spirit, toughness, and nonacceptance. Thus, by addressing the wider relevance of coping styles assessed by the SICQ throughout the trajectory to recovery of critically ill patients, the understanding of the psychological coping of critically ill patients may be increased.

## Interpretation

Adaptive coping styles measured with the SICQ were associated with long-term mental HRQoL, hospital length of stay, and 12-month mortality among critically ill patients. Further research is needed to understand the clinical role of monitoring and intervening on coping styles as assessed with the SICQ.

## Supplementary Data

e-Online Data
