# Supplementary material for: SICQ Coping and the Health-Related Quality of Life and Recovery of Critically Ill ICU Patients: A Prospective Cohort Study
Source: Chest. 2021 Jun 26;161(1):130–9. doi: 10.1016/j.chest.2021.06.033 (PMC8783033; doi:10.1016/j.chest.2021.06.033)
Supplement: e-Online Data [file mmc1.pdf]

CHEST Online Supplement

SICQ Coping and the Health-Related Quality of Life and Recovery of Critically Ill ICU Patients

A Prospective Cohort Study

Edwin J. Boezeman, PhD; José G.M. Hofhuis, RN, PhD; Christopher E. Cox, MD, MPH; Reinout E. de Vries, PhD; and Peter E. Spronk, MD, PhD, FCCP

CHEST 2022; 161(1):130-139

Online supplements are not copyedited prior to posting and the author(s) take full responsibility for the accuracy of all data.

© 2021 AMERICAN COLLEGE OF CHEST PHYSICIANS. Reproduction of this article is prohibited without written permission from the American College of Chest Physicians. See online for more details. **DOI:** 10.1016/j.chest.2021.06.033

CHEST Online Supplement

e-Table 1. Correlations between Proxy-recorded SICQ-coping and Patient HRQoL

	Patients' mental HRQoL recorded:			
	At hospital discharge	3 months	6 months	12 months
(*n* = 133 proxies)		after discharge	after discharge	after discharge
SICQ-coping proxy recorded pre-ICU				
Positivism	.31*** (.29*;.28*;.32*)	.38*** (.36*;.38*;.39*)	.24* (.17; .23*;.24*)	.17 (.14;.17;.20)
Redefinition	.13 (.06;.07;.14)	.15 (.13*;.14;.14)	.06 (.02;.05;.06)	.04 (.02;.02;.03)
Toughness	.07 (.07;.04;.10)	-.09 (-.11;-.09;-.05)	-.01 (-.05;-.02;.02)	.04 (.01;.03;.09)
Fighting Spirit	.19 (.16;.11;.19)	.25** (.23*;.24*;.25*)	.10 (.07;.08;.11)	.21* (.19;.19;.22*)
Non-Acceptance	.14 (.12;.04;.13)	-.07 (-.07;-.10;-.08)	.07 (.09;.04;.06)	.05 (.06;.02;.05)

	Patients´ physical quality of life, recorded:			
	At hospital discharge	3 months	6 months	12 months
(*n* = 133 proxies)		after discharge	after discharge	after discharge
SICQ-coping proxy recorded pre-ICU				
Positivism	.03 (.01;-.04.;.03)	.16 (.14;.14;.17)	.23* (.17;.21*;.23*)	.08 (.03;.05;.10)
Redefinition	.24* (.20*;.17;.24*)	.23* (.20*;.19;.23*)	.25** (.20*;.21*;.25*)	.23* (.17;.18;.23*)
Toughness	.04 (.06;-.00;.05)	.04 (.02;.02;.03)	.08 (.05;.05;.09)	-.01 (-.04;-.06;.04)
Fighting Spirit	-.00 (-.01;-.11;-.00)	-.01 (-.05;-.09;-.01)	.14 (.12;.08;.14)	.04 (.01;-.06;.05)
Non-Acceptance	.23* (.24*;.13;.23*)	-.12 (-.12;-.21*;-.11)	-.06 (-.05;-.13;-.06)	.04 (.04;-.05;.04)

Note. * *P* < .05, ** *P* < .01, *** *P* < .001; Coefficients between parentheses are partial correlation coefficients controlling for respectively the patients' severity of illness – score (APACHE), patients' age, and patients' gender type (0 = male, 1 = female)

Online supplements are not copyedited prior to posting and the author(s) take full responsibility for the accuracy of all data.

e-Table 2. Spearman correlations (Rho) between SICQ-coping and days of stay

(*n* = 133 proxies)	Patients' No. of hospital days	Patients' No. of ICU days
SICQ-coping patient recorded pre-ICU		
Positivism	-.21*** (-.15**;-.20***;-.22***)	-.16*** (-.08;-.14**.;-.15**)
Redefinition	-.08 (-.06;-.07;-.08)	-.03 (.02;-.02;-.03)
Toughness	-.06 (-.05;-.05;-.06)	-.01 (-.01;-.00;-.01)
Fighting Spirit	-.05 (-.06;-.05;-.06)	-.03 (-.05;-.02;-.03)
Non-Acceptance	.01 (.05;.03;.01)	.09 (.13*;.11*;.09)
SICQ-coping proxy recorded pre-ICU		
Positivism	-.29*** (-.22*;-.25**;-.29***)	-.17 (-.06;-.14;-.17)
Redefinition	-.25** (-.20*;-21*;-.25*)	-.17 (-.11;-.12;-.17)
Toughness	-.03 (-.04;-.02;.03)	.00 (.00;.01;.02)
Fighting Spirit	-.16 (-.15;-.12;-.16)	-.08 (-.04;-.04;-.08)
Non-Acceptance	-.08 (-.09; -.02;-.09)	.06 (.07;.12;.06)

Note. * *P* < .05, ** *P* < .01; Coefficients between parentheses are partial correlation coefficients (Rho) controlling for respectively the patients' severity of illness – score (APACHE), patients' age, and patients' gender type (0 = male, 1 = female)

Online supplements are not copyedited prior to posting and the author(s) take full responsibility for the accuracy of all data.

e-Table 3. Cox regression survival analysis with death as event

Predictor	B	SE	Wald	*P*-value	HR	CI 95% HR
Patient pre-ICU						
(n = 391 patients)						
Positivism	-.57	.19	9.22	.002	0.57	0.39 to 0.82
Redefinition	-.21	.17	1.49	.22	0.81	0.58 to 1.14
Toughness	-.09	.18	0.28	.59	0.91	0.65 to 1.28
Fighting Spirit	.03	.21	0.02	.90	1.03	0.68 to 1.55
Non-acceptance	-.27	.19	2.02	.16	0.77	0.53 to 1.11
Proxy pre-ICU						
(n = 133 proxies)						
Positivism	-.90	.40	5.06	.02	0.41	0.19 to 0.89
Redefinition	-.30	.38	0.59	.44	0.75	0.35 to 1.58
Toughness	.48	.40	1.42	.23	1.61	0.74 to 3.53
Fighting Spirit	.00	.43	0.00	.99	1.00	0.43 to 2.33
Non-acceptance	-.00	.40	0.00	.99	1.00	0.46 to 2.16

Note. The ‘event’ outcome variable death (no/yes) involved whether the patient had deceased during the study, the ‘time’ variable was the number of days in the study

Online supplements are not copyedited prior to posting and the author(s) take full responsibility for the accuracy of all data.

e-Table 4. Logistic regression analysis results with location of stay after hospital discharge as outcome

Predictor	B	SE	Wald	*P*-value	OR	CI 95% OR
Patients' pre-ICU						
(n = 391 patients)						
Positivism	.48	.20	6.04	.01	1.62	1.10 to 2.37
Redefinition	.06	.18	0.10	.76	1.06	0.74 to 1.51
Toughness	.23	.19	1.44	.23	1.25	0.87 to 1.81
Fighting Spirit	.38	.21	3.33	.07	1.47	0.97 to 2.22
Non-acceptance	-.11	.18	0.35	.56	0.90	0.63 to 1.28
Constant	-2.61	1.11	5.58	.02	0.07	-
Proxy' pre-ICU						
(n = 133 proxies)						
Positivism	.26	.28	0.87	.35	1.13	0.75 to 2.26
Redefinition	.06	.26	0.05	.83	1.06	0.63 to 1.77
Toughness	-.08	.27	0.08	.78	0.93	0.55 to 1.57
Fighting Spirit	.18	.32	0.32	.57	1.20	0.64 to 2.25
Non-acceptance	.42	.29	2.19	.14	1.53	0.87 to 2.68
Constant	-1.31	1.47	0.80	.37	0.27	-

Note. The dichotomous outcome variable 'location of stay after hospital discharge' involved the location to which the patient was transferred to after hospital stay and this variable was scored as 'nursing home or rehabilitation center' (low score) or 'home' (high score).

Online supplements are not copyedited prior to posting and the author(s) take full responsibility for the accuracy of all data.

CHEST® Online Supplement

Online supplements are not copyedited prior to posting and the author(s) take full responsibility for the accuracy of all data.
